# Fabrication of a New, Low-Cost, and Environment-Friendly Laccase-Based Biosensor by Electrospray Immobilization with Unprecedented Reuse and Storage Performances

**DOI:** 10.1021/acssuschemeng.1c07604

**Published:** 2022-01-24

**Authors:** Mattea Carmen Castrovilli, Emanuela Tempesta, Antonella Cartoni, Paolo Plescia, Paola Bolognesi, Jacopo Chiarinelli, Pietro Calandra, Nunzia Cicco, Maria Filomena Verrastro, Diego Centonze, Ludovica Gullo, Alessandra Del Giudice, Luciano Galantini, Lorenzo Avaldi

**Affiliations:** † Istituto di Struttura della Materia-CNR (ISM-CNR), Area della Ricerca di Roma 1, 00015 Monterotondo, Italy; ‡ CNR-Institute of Environmental Geology and Geoengineering (CNR-IGAG), Area della Ricerca Roma1, Via Salaria km 29.300, 00015 Monterotondo, Italy; § Department of Chemistry, Sapienza University, P.le Aldo Moro 5, 00185 Roma, Italy; ∥ CNR-Institute for the Study of Nanostructured Materials (CNR-ISMN), Area della Ricerca Roma1, Via Salaria km 29.300, 00015 Monterotondo, Italy; ⊥ CNR-Institute of Methodologies for Environmental Analysis (CNR-IMAA), Contrada Santa Loja, Tito Scalo, 85050 Potenza, Italy; # Istituto di Struttura della Materia-CNR (ISM-CNR), Contrada Santa Loja, Tito Scalo 85050, Potenza, Italy; ∇ Dipartimento di Scienze Agrarie, degli Alimenti e dell’Ambiente, Università degli Studi di Foggia, via Napoli, 25, 71122 Foggia, Italy

**Keywords:** reuse, storage performance, immobilization, electrospray deposition, biosensor, laccase, catechol detection

## Abstract

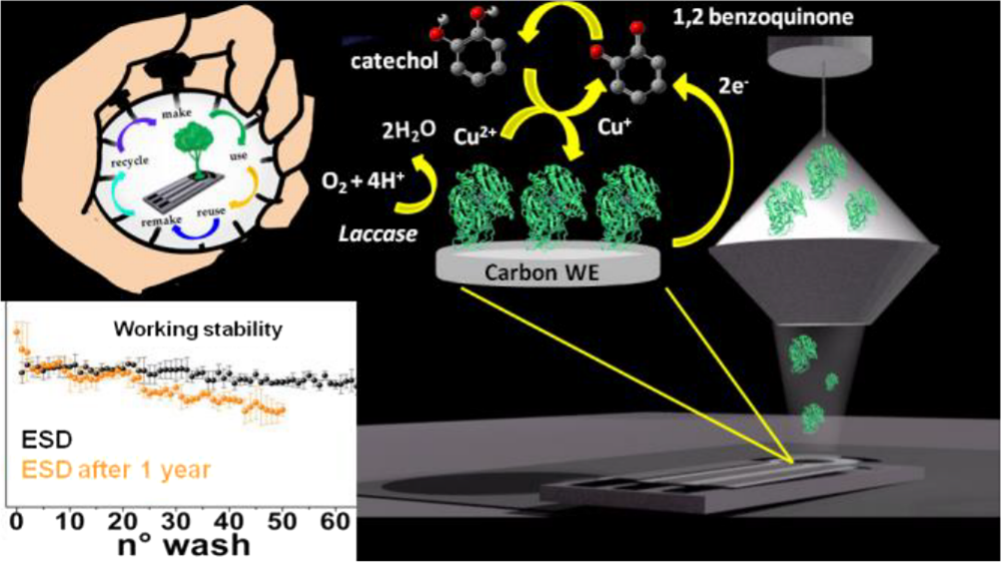

The fabrication of enzyme-based biosensors has received much attention for their selectivity and sensitivity. In particular, laccase-based biosensors have attracted a lot of interest for their capacity to detect highly toxic molecules in the environment, becoming essential tools in the fields of white biotechnology and green chemistry. The manufacturing of a new, metal-free, laccase-based biosensor with unprecedented reuse and storage capabilities has been achieved in this work through the application of the electrospray deposition (ESD) methodology as the enzyme immobilization technique. Electrospray ionization (ESI) has been used for ambient soft-landing of laccase enzymes on a carbon substrate, employing sustainable chemistry. This study shows how the ESD technique can be successfully exploited for the fabrication of a new promising environment-friendly electrochemical amperometric laccase-based biosensor, with storage capability up to two months without any particular care and reuse performance up to 63 measurements on the same electrode just prepared and 20 measurements on the one-year-old electrode subjected to redeposition. The laccase-based biosensor has been tested for catechol detection in the linear range 2–100 μM, with a limit of detection of 1.7 μM, without interference from chrome, cadmium, arsenic, and zinc and without any memory effects.

## Abstract 2

ESD as an environment-friendly immobilization engineered technique to fabricate metal-free laccase-based biosensors with long-term operation and stability, suitable for recycling.

## Introduction

The fields of green chemistry and white biotechnology look at biocatalysts as cutting-edge technology, thanks to their ability to exploit the selectivity and low energy requirements of enzymes to create nontoxic biosensing devices. In the construction of a successful performing enzymatic biosensor, many fundamental factors must be taken into consideration.^1^ Among them, the choice of the correct immobilization method of the bioreceptor on the surface of the transduction system is considered a crucial one.^2,3^ The immobilization procedure must preserve the maximum activity of the bioreceptor and improve the performance of the device in terms of storage and reuse, the latter being mandatory in order to reduce the pollution due to disposable devices. The immobilization procedure is capable of facilitating the recycling of enzymes, allowing a reduction in the cost of the biosensor production process by up to 50%.^4^

An enzyme could undergo changes in its physical and chemical properties upon immobilization, depending on the choice of the immobilization method. Thus, the maintenance of the catalytically active structure is a key factor to maximize the stability and reactivity of the enzyme in its immobilized state.^5−10^

Up to now, the two main strategies exploited to construct highly sensitive laccase-based biosensors are the hard-working covalent attachment of the bioreceptor on the surface and the physical adsorption. In both the cases, poor results have been achieved in terms of stability in time and storage, the latter having the further drawback to be carried out at 4 °C.^2,5^

Here, we demonstrate how the use of the electrospray deposition (ESD) technique to perform ambient soft-landing immobilization can be employed for the production of a durable and reusable low-cost laccase-based biosensor. The electrospray ionization (ESI) technique^11,12^ has aroused considerable interest, thanks to its ability to generate sprays of charged monodispersed nanoaggregates, which can be delivered to surfaces with very low kinetic energies (soft-landing), producing thin and uniform coating films of fine particles.^13−16^

The ESD technique is based on the use of a low-concentration solution of the molecule of interest flowing in a small capillary held at a high voltage (typically a few kV) with respect to a grounded counter electrode a few mm away.^17,18^ At the tip of the emitter, the surface tension of the liquid competes with the effect of the high electric field. When the latter balances the surface tension, the so-called “Taylor cone” is formed. Inside the cone, the Coulomb explosion creates a spray of charged droplets. The size of the droplets, in some cases down to nanometers, continues to decrease as the solvent evaporates and, at the end, a gas of molecular ions is formed.^19,20^ This approach provides effective deposition of the molecules that can also be carried out at ambient pressure or in a controlled atmosphere, with a significant reduction in the cost and time of the process compared to those in vacuum techniques. Furthermore, the ESD process that can be easily automated requires a very small amount of material to be sprayed, making deposition possible in safe, compact, and portable devices. ESD saw its first application in nuclear physics to fabricate a thin layer of radioactive material as a source of high-energy particles (α or β).^12,21−23^ Later, the method was applied to molecules, over a wide range of molecular weights, for example, low-weight molecules, synthetic polymers, proteins, and DNA.^17,18,24−29^ Thus, ESD has been employed in the formation of layers of semiconductive ceramics such as metal-oxide films,^30^ modification of silicon surfaces with layers of silk-forming peptides to enhance the adhesion of living cells, preparation of DNA and protein samples for scanning tunneling microscopy,^31^ formation of protective polymer coatings on electrode surfaces,^25^ as well as applications for biosensors and biochips (e.g., protein-/DNA-microarray and microfluidic devices),^18,32^ antifouling or biocompatible coatings for medical devices, high-performance filter media,^33^ biomaterial scaffolds for tissue engineering,^34−36^ nanotechnology, and nanoelectronics.^19^ Moreover, the combination of high-flux ESI sources with mass spectrometric selection in vacuum led to the deposition of polyatomic ions with well-defined composition, charge states, and kinetic energy to prepare controlled interfaces for applications in energy storage, catalysis, soft materials, and biology.^15,37,38^ Among all these applications, ESD has also been used to prepare surfaces with ceramic, nanoparticles, or polymer coatings designed to accept bioactive species or to inhibit bacterial adhesion to enhance cell growth^39^ and to immobilize proteins for in situ analysis with other techniques,^35,40^ as well as to write two-dimensional (2D) metallic nanostructured patterns for surface-enhanced Raman spectroscopy using silver nanoparticles.^41,42^

In this work, we demonstrate the feasibility of using the ESD technique at room temperature and atmospheric pressure for the direct soft-landing deposition of bioactive molecules on unmodified commercial carbon screen-printed electrodes (C-SPE) and describe all the steps needed to produce a coating of bioactive species that can be used in the manufacturing of electrochemical biosensors. The bioactive species chosen is the laccase enzyme from Trametes Versicolor (EC 1.10.3.2), which is considered the most suitable “green catalyst” enzyme requiring only oxygen molecules as reactants and producing only water molecules as byproducts.^43^ This enzyme belongs to the oxidoreductase class of enzymes with the molecular weight in the range 60–100 kDa, synthesized by plants, fungi, some bacteria, and insects;^44^ thanks to its catalytic activity and the wide range of substrates, it can be used in various fields of industrial applications from bioremediation to environmental and agri-food. However, as stated by Alvarado-Ramírez et al.^2^ “*Despite all the advantages of using laccases, some disadvantages include lack of long-term operation, stability, or inability to recover the enzyme, making it impossible to use laccase at an industrial scale*”. This work paves the way to overcome these limitations by employing soft-landing deposition as immobilization.

In 2020, Castrovilli et al.^45^ have presented a laccase-based biosensor fabricated by the ESD set up using the procedures described in detail for the first time here. This biosensor, which uses a carbon black-modified electrode via drop-casting, shows good anchorage stability of laccase and near 100% retention of its performances up to 25 washes without any enzyme leaching. In the following, we will present a new untreated laccase-based carbon biosensor (eLac-C-SPE) that, reaching stability up to 63 washes (twice the washes of the previous biosensor^45^) and demonstrating an unprecedented reuse feature, exceeds the performances of the previous one and makes this new device a low-cost, environment-friendly, and economically sustainable option.

The retaining of enzyme activity after ESD immobilization, as well as the analytical performances of the manufactured biosensors in terms of working and storage stability, the limit of detection, the linear range of the amperometric response, repeatability, sensitivity, selectivity, and accuracy have been demonstrated. The details of the studies to find the optimal operative conditions to fabricate the biosensor are reported in the Supporting Information.

## Materials and Methods

### Chemicals and Instrumentation

Fungal laccase from *Trametes versicolor* (TvL) (E.C. 1.10.3.2, activity: 0.5 U/mg), ethanol (99.8%), methanol (99.8%), formic acid (95%), syringaldazine (98%), and catechol (99%) were purchased from Sigma Aldrich (Merck Group) and used as provided by the company. All the solutions were prepared using double-distilled water (Milli-Q system, Millipore). The catechol was used in 1 mM solution with citric acid/sodium citrate buffer 0.1 M at pH 4.5 for amperometric measurements. The study of the percentage of the organic solvent to be used for the spray solution has been performed using a citric acid/sodium citrate buffer 0.1 M at pH 4.5, following the syringaldazine assay. The electrochemical measurements were performed using the portable potentiostatPalmSens4 (Palm Instrument, The Netherlands). The spectrophotometric measurements were performed using a Jasco ultraviolet–visible (UV–vis) V660 double-beam spectrophotometer. The image of the deposit on the C-SPE was acquired using the Olympus IX53 Microscope and the Malvern Panalytical Morphologi 4-ID. The small-angle X-ray scattering (SAXS) measurements were performed at SAXSLab Sapienza with a Xeuss 2.0 Q-Xoom system (Xenocs SA, Sassenage, France) equipped with a microfocus GeniX 3D X-ray Cu source (λ = 1.5419 Å) and a two-dimensional (2D) Pilatus3 R 300K detector placed at a variable distance from the sample (Dectris Ltd., Baden, Switzerland). The amount of laccase deposited was quantified using a custom quartz crystal microbalance (QCM) manufactured at the Istituto di Inquinamento Atmosferico (IIA) of the CNR, Area di Ricerca di Roma 1 in Montelibretti (Rome, Italy).^46,47^ The screen-printed electrodes used for the tests of deposition were the Metrohm DropSens screen-printed carbon electrodes DRP-110 (C-SPEs) with carbon working (4 mm diameter) and counter electrodes, and an Ag reference electrode.

### Electrospray Deposition Setup

**Figure 1:**
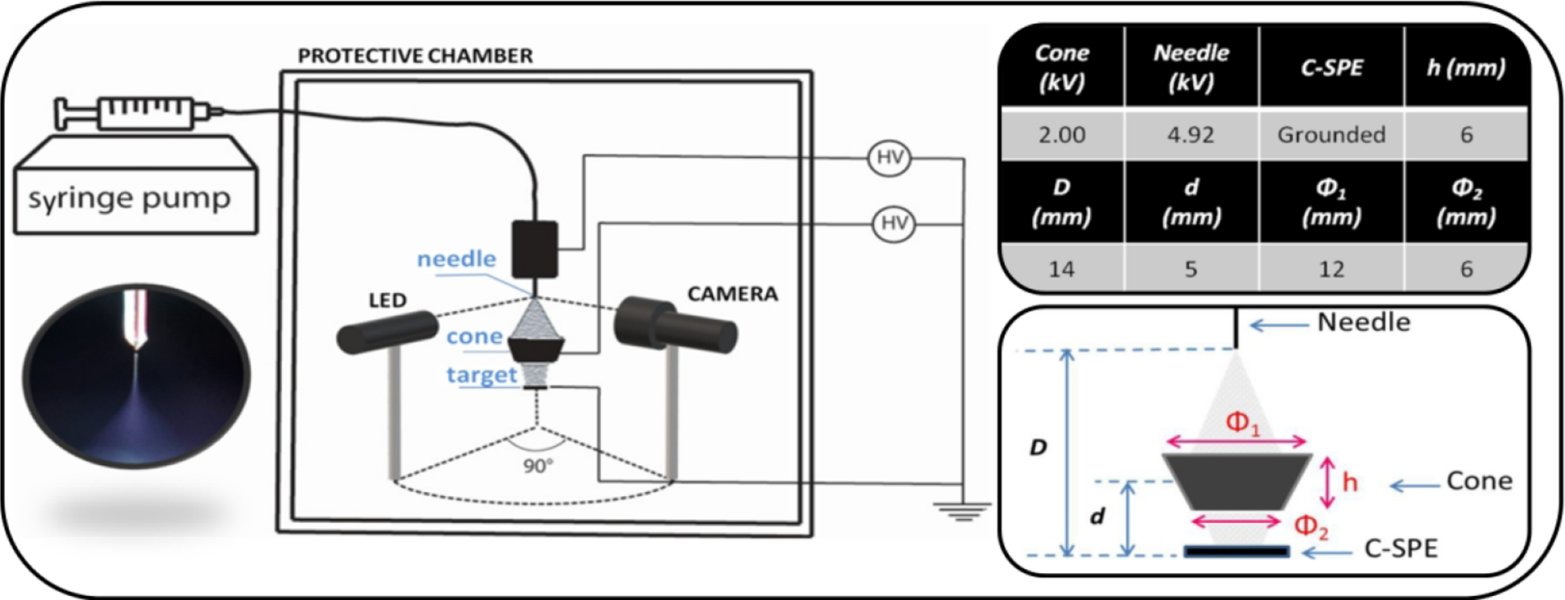
Schematic of the ESD setup and a picture of the Taylor cone generated during deposition (on the left); table of the voltages and geometric parameters in the ESD process and an enlarged scheme of the deposition region (on the right).

The ESD setup is shown in Figure 1, where the counter electrode (target) at ground is replaced by a C-SPE.

The entire setup is located in a protected environment at ambient pressure and temperature in order to avoid jet fluctuations. The stability of the jet, illuminated by an LED, was monitored in real time during the entire deposition by means of a 6 × 16 mm 10° monocular (SPECWELL) coupled to a BRESSER MikrOkular Full HD digital camera. The LED and the camera were positioned at right angle to achieve the best contrast of the image of the cone, as shown in the inset of Figure 1, in which the characteristic shape of the Taylor cone^48^ is visible. The instrumentation consists of a Pump 11 Elite infusion (Harvard Apparatus) equipped with a Hamilton syringe (250 μL total volume), which is connected to a silica capillary (300 μm internal diameter) ending with a steel needle (100 μm inner diameter), where a high voltage is applied. Between the needle and the target, a focusing electrode (cone) has been added. This electrode changes the spatial electric field between the needle and the counter electrode and prevents electric disturbances. It also improves the control of the deposition area in the process.^49^

The alignment between the spray needle, the focusing cone electrode, and the target is a crucial parameter in deposition. Moreover, the distance between these three elements can be influenced by the composition of sprayed solution.^50^ In the insets of Figure 1, a schematic description with typical values of distances and applied voltages is shown. Once the voltages were fixed, a series of focusing cones of different shapes and sizes was tested in order to find the conditions for continuous and uniform deposition over the entire area of the SPE working electrode (see Table S1 in the Supporting Information). In order to quantify the amount of enzymes deposited with the different cones as a function of the deposition time and to connect these quantities to the activity of the deposited laccase, these depositions were carried out on a quartz microbalance. With respect to our previous work,^45^ here, the choice of a focusing electrode with a larger inlet diameter (Φ_1_ = 12 mm) and a shorter distance between the cone and the needle (6 mm compared to 9 mm of the previous work) favors a better definition of the spray and the formation of a more uniform layer of laccase. The overall distance of the substrate from the needle is shorter in this setup. This is about 13 mm because the commercial C-SPE has a thickness of 1 mm. In the previous work,^45^ home-made electrodes printed on a polyester sheet with a thickness of about 100 μm were used. Moreover, the area of graphite deposition in the C-SPE is larger (4 mm diameter) compared to the carbon black SPE (3 mm diameter) and has to be considered more uniform. Indeed, the manufacturing procedure of the home-made carbon black electrodes involved the modification of the graphite surface by drop-casting a solution of carbon black nanoparticles. As it is known,^51^ the drop-casting procedure has no control in the shape of the film produced, and this may have led to the formation of a coffee-ring of carbon black nanoparticles, making the surface exposed to the deposit of the laccase no longer uniform. The details of the studies performed to identify the optimal deposition parameters are described in Section 1 of the Supporting Information.

### Laccase Preparation for Ambient Soft-Landing Immobilization by Electrospray

The stock solution of laccase was prepared by diluting Sigma-Merck-lyophilized laccase in 5 mL of MilliQ water to a final concentration of 5 μg/μL and split into Eppendorf tubes maintained at −18 °C. For the preparation of the working solution, the stock solution was diluted to 2 μg/μL at 20% of methanol in water (solution A in Table S2 of the Supporting Information). This procedure avoids temperature degradation of the sample, and all tests use a fresh solution of equal concentration. The steps followed to find this working solution for the ESI spray and the best pH buffer for the amperometric analysis are detailed in Section 2 and subsections 2.1 and 2.2 of the Supporting Information.

### SAXS Characterization of Laccase in Solution

Once the best solvent composition to be sprayed was chosen, a SAXS characterization of the enzyme dispersion in the solution was performed. The sample was loaded in a thermalized vacuum-tight quartz capillary cell, and the measurements were performed at 25 °C at three different sample-detector distances, in order to record the sample scattering within the scattering vector range of 0.005 < *q* < 1 Å^–1^ (*q* = 4πsin(θ)/λ, where 2θ is the scattering angle). 2D scattering patterns were collected and subtracted for the “dark” counts. The images were then masked, azimuthally averaged, and normalized for the transmitted beam intensity, exposure time, and subtended solid angle per pixel, using the FoxtTrot software, version 3.4.9 (developed by Soleil Synchrotron and Xenocs SAS). The results of subsequent 1800 s exposures were averaged since superimposable. The one-dimensional intensity vs *q* profiles were then subtracted for the contributions of the solvent and of the capillary and put in absolute scale units (cm^–1^) by dividing for the capillary thickness. Model-independent data interpretation and the calculation of scattering profiles from atomic models were performed using the tools of the ATSAS package,^52^ and additional fits with analytical model intensities were obtained with the SasView software.^53^

### Effect of the ESI Process

The effect of the ESI process on the enzyme activity has been investigated by syringaldazine assay on laccase dissolved after deposition.

A 2 μg/μL laccase solution with 20% of methanol was electrosprayed for 30 min at 1 μL/min and deposited on an aluminum foil after removing the focusing cone to avoid a partial loss of the sprayed material on the walls of the cone. The laccase was then dissolved again using 1350 μL of buffer solution and its activity compared with the one of test *tA* (Table S2 of the Supporting Information) to determine the amount of activity loss due to the electrospray process. The measurement has been repeated three times. See Section 2.3 of the Supporting Information.

### Choice of Deposition Time and Focusing Cone

The deposition time is strictly related to the amount of enzyme to be deposited on the electrode. For the purpose, the ability to control the deposition area by focusing the spray via an additional electrode (Figure 1) and to assess the amount of enzymes deposited by means of a QCM has been investigated.

Five conical electrodes of different heights and widths have been prepared and tested. The diameter of the spot of the deposited material in each geometry has been measured and reported as Φ_deposit_ (Table S1 in the Supporting Information). The C1 cone with *h* = 6 mm, Φ_1_ = 12 mm, Φ_2_ = 6 mm, and Φ_deposit_ = 4.5 mm has been chosen and used for all the subsequent depositions. Then, the QCM has been used to measure the amount of the deposited laccase for each deposition time, correlating this quantity to the amperometric response of the electrode (see Section 2.4 of the Supporting Information).

### Electrochemical Characterization of the Electrosprayed Laccase on Screen-Printed Carbon Electrodes

Electrochemical experiments were carried out at room temperature by amperometric analysis with an applied potential of −0.03 V vs Ag reference electrode in a total volume of 100 μL, recording the current signals every 0.5 s. The study of the laccase activity was performed using a 50 μM final concentration of catechol and 0.1 M citric acid/sodium citrate buffer at pH 4.5. The laccase mechanism for catechol detection is based on the electrocatalysis of catechol oxidation to its corresponding 1,2 benzoquinone, which is coupled with the electrocatalytic reduction of dioxygen to water on the working electrode surface. The biosensor response was expressed as the difference between the analyte and the background current signals. The depositions on C-SPE were carried out for a period of 30 min at a flow rate 1 μL/min, using the focusing cone C1 and the solution A for the spray (Tables S1 and S2 in the Supporting Information).

### Catechol Detection in Real Water Samples

Three water samples were tested: lake and well water from countryside north of Rome (Lazio, Italy) and undrinkable tap water from CNR Research Area of Roma 1. These samples were diluted 1:2 with 0.2 M buffer solution of citric acid/sodium citrate at pH 4.5 and loaded onto the sensor for the analysis, without any pretreatment.

## Results and Discussion

### SAXS Characterization of Laccase in ESI Solution

**Figure 2:**
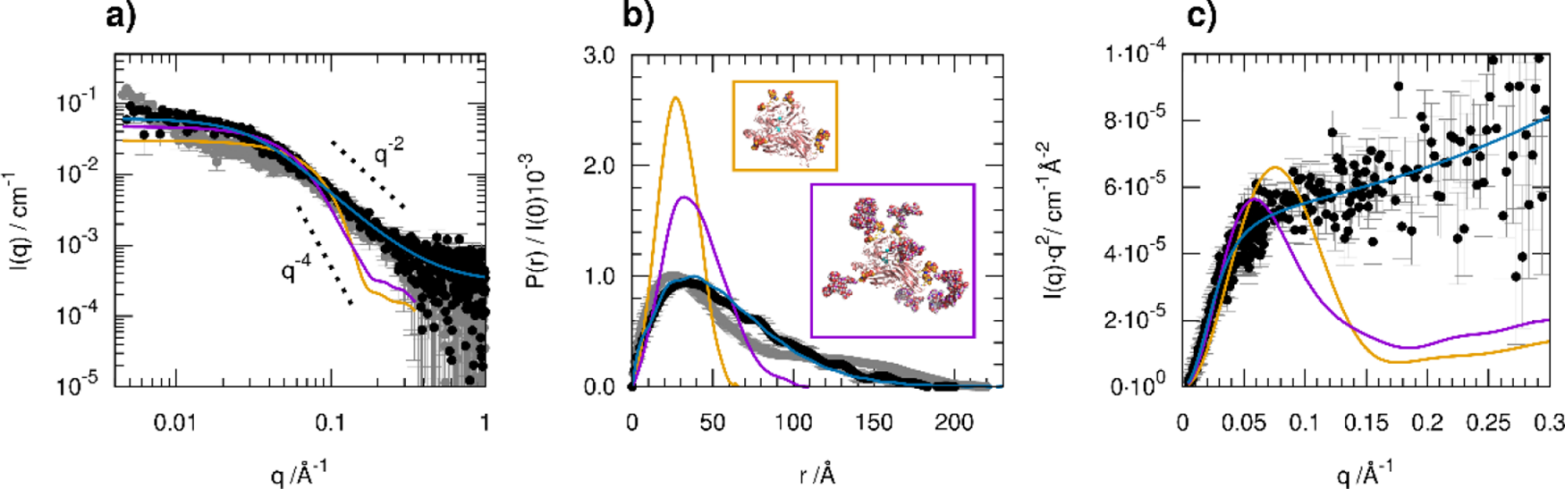
SAXS characterization of laccase in ESI solution: (a) experimental scattering profiles (dots) and model intensities. (b) Pair distance distribution functions calculated from the scattering patterns shown in (a) by the application of the indirect Fourier transform. In the insets, the structure of the atomic models (PDB entry 1GYC, orange frame, and a further glycosylated structural model, purple frame) considered for calculating theoretical scattering profiles of laccase are shown in ribbon representation (protein chain) and as a sphere (glycan chains). (c) “Kratky plot” representation of the scattering data and model intensities.

The SAXS profile of a freshly prepared dispersion of laccase 2 μg/μL in a water–methanol mixture with 20% methanol volume content (solution A) is shown in Figure 2a (black dots), together with an additional profile collected on the aged suspension after 1 week (gray dots). The indirect Fourier transform method applied to the data provided a pair distance distribution function (Figure 2b), indicating that the dispersed enzyme particles have overall a maximum size of 150 ± 10 Å and an average radius of gyration *R*_g_ of 49 ± 3 Å. After 1 week, the SAXS intensity in the very first points underwent a slight upturn, whereas the profile in the higher *q* range slightly decreased, suggesting that the enzyme particles underwent some aggregation with the formation of larger clusters up to a maximum distance of 200 Å.

We first compared the collected data with the theoretical SAXS profile calculated from the crystallographic structure of the laccase from Trametes versicolor available in the Protein Data Bank (PDB) entry 1GYC^54^ (orange solid lines in Figure 2). This structure, which includes a total of six glycosylation sites with three monosaccharide and three disaccharide units covalently attached to Asn residues, would predict a much smaller particle size with an *R*_g_ value of 22 Å and a maximum size of 70 Å. This finding is not surprising, knowing that the active laccase enzyme is found as a rather heterogeneous mixture of variably glycosylated forms, an aspect that had hampered in the past the production of crystals for structural studies.^54^ A model with a higher degree of glycosylation was constructed from this crystal structure using the appropriate tool of the ATSAS package (glycosylation) and imposing eight known glycosylation sites according to the UniProt annotations,^55^ and using the heaviest glycan chains available in the database, with a mass of approximately 2000 Da per chain. The resulting model (purple solid lines in Figure 2) would have an *R*_g_ value of 31 Å and a maximum size of 110 Å, suggesting that the presence of relatively long glycan chains attached at the enzyme glycosylation sites could partially explain the larger dimensions obtained from the SAXS data. The possible formation of protein–protein oligomers could also be considered to account for the observed average size. We notice that the experimental maximum distance (150 Å) is roughly twice the calculated size from the PDB structure (70 Å). However, there is an additional aspect deduced from the scattering profile, which could not be described using a static structural model, even with an increased size because of glycosylation or dimerization. In the intermediate *q* range (0.05–0.5 Å^–1^), the data showed a characteristic slope close to *q*^–2^, which is characteristic of flexible polymeric chains, whereas a compact globular protein would tend to the slope expected for particles with a well-defined surface, according to the Porod law (*q*^–4^) (black dotted lines in Figure 2a). This difference is highlighted when plotting the data as *I*(*q*)·*q*^2^ vs *q* (“Kratky plot,” Figure 2c), a representation in which the globular behavior is associated with a bell-shaped profile, whereas a flexible chain-like behavior is associated with a plateau or linear increase. The SAXS data, therefore, suggest that a notable contribution to the scattering is given by flexible chains, and indeed, the scattered intensity calculated from the analytical model for a random coil with an *R*_g_ value of 47 Å can reasonably reproduce the data (blue lines in Figure 2). This contribution could be probably related to a soluble fraction of polysaccharide molecules that are present in the enzyme product, either free or attached as glycan chains to the enzyme, which could play an interesting role in the formation of the stable layer in which the active enzyme is immobilized on the sensor surface after ESI deposition, as characterized in the following sections.

### Effect of the ESI Process

The study of the effect of the ESI process on laccase activity, described in detail in Section 2.3 of the Supporting Information, shows a preserved activity of 70% after deposition. This result demonstrates that, overall, the ESD process performed with the described procedure is a promising technique.

On the basis of the present and literature data, some hypotheses can be put forward to explain the decrease in activity. Modifications in the pH,^56^ solvent,^57,58^ and temperature^59^ can affect the configuration of the proteins, with a consequent alteration of the polar and nonpolar interactions that stabilize the protein and can result in denaturation in the solution phase. Even if correlations between charge-state distributions and solution-phase structures have been found, it cannot be proved rigorously that a gas-phase structure corresponds to a solution-phase structure. Moreover, during electrospray, the high potential of the needle can change the solution-phase environment, somehow affecting both the solution- and gas-phase structures. Then, in the gas phase, the Coulomb forces between the adducted charges may become much more important and modify the gas-phase structure.^60^

Malinowski and co-workers,^61^ using a soft plasma jet deposition technique, found a decrease in the laccase activity of 43.9 and 57% passing from 3 to 4 and 5 kV, respectively, and attributed this to a change in the secondary structure induced by the applied high voltage. In our case, when a voltage of 4.9 kV is applied, the activity decreases by about 30%.

Because of high voltage, hydroxyl radicals (OH^•^) and H_2_O_2_^62^ could be present in the nebulized solution, which trigger ion-/radical-molecule reactions.^63−68^ It is known^69,70^ that such radicals can react with amino acids such as cysteine, aromatic rings of phenylaniline, tyrosine, and tryptophan, causing a reduction in the alkaline phosphatase activity by the degradation of the aromatic rings.^71^

Crystallographic studies of laccase show that the active center contains histidine residues linked to the Cu atoms. The consequent degradation of histidine imidazole rings, because of the active species, may imply a lowering of the enzyme activity. It may be possible that, in our conditions, ionization of the air near the tip of the needle generates different species that can interact with the enzymes. Takaj et al. in 2012^72^ showed O_3_ as the molecule responsible for the increase in the α-helix substructure in lysozyme after plasma treatment.

To speculate on the possible mechanisms of biocoating formation, we could assume the starting hypotheses of Malinowski et al.^61^ that only external amino acids in the laccase enzyme structure take part in cross-linking and bonding reactions. This could allow the retention of the active center structure of the molecule in its unchanged form. Nevertheless, we cannot ignore the contribution to the cross-linking between the laccase layers because of the polysaccharide chains found through the SAXS analysis. Following the assumption of Malinowski et al.,^61^ in addition, at the initial stage of deposition, cross-linking between laccase molecules occurs through a radical reaction, leading to amide bond formation. Continuing deposition, the most external amino acid residues of the enzyme could be responsible for the creation of amide bonds between amine and carboxyl groups of the amino acids of different monomers and layers, resulting in cross-linking between the layers leading to a final amorphous film. The good robustness in working stability could be ascribed to the removal, wash after wash, of the outermost layers of the deposit, which leaves active layers of laccase at each reuse.

### Study of Deposition Time

**Figure 3:**
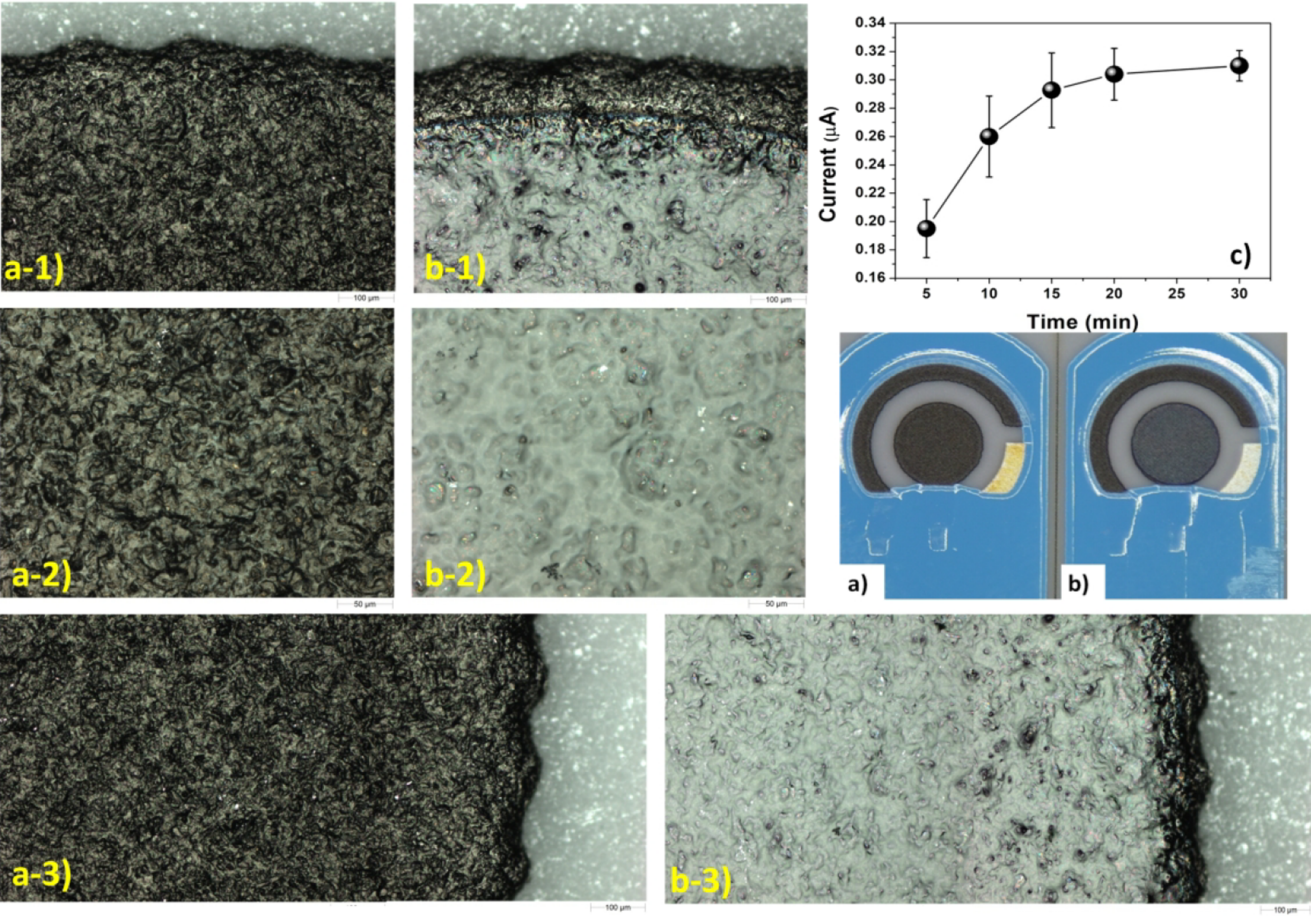
Images of pristine (a) and modified (b) C-SPE working electrode. (a-1, a-2, a-3) and (b-1, b-2, b-3) are the magnification of the top, central, and lateral parts of pristine and modified C-SPE working electrodes, respectively. (c) Amperometric measurements of the laccase–catechol system for laccase deposited on C-SPE at different deposition times using solution A (Table S2), C1 cone, and the geometry reported in Figure 1. *n* = 4 for each deposition time with buffer pH 4.5 and a catechol concentration of 50 μM.

The images in Figure 3 show how using the focusing cone C1 and the parameters described in Figure 1, the diameter of the spot precisely fits the diameter of the working electrode and, at a first sight, it seems quite uniform.

The results of the study reported in Figure S5 of the Supporting Information highlight that a constant deposition rate is maintained over time. The time of deposition directly determines not only the amount of the deposited laccase on the sensor, but also the interaction time of the deposited laccase molecules with active species present in the incoming spray. However, the amperometric measurements (Figure 3c) of the laccase activity on the electrode at increasing deposition times show that the mean current reaches a plateau value of 0.30 ± 0.02 μA in about 15 min and then it varies no more. This result may be due to (i) the molecular damage and deactivation of previously deposited laccase through bombardment by active species such as ions, radicals, and molecular fragments^73^ and/or (ii) the degree of laccase cross-linking in the deeper layers that hamper both the approach to the active site by catechol and the reaching of the working electrode surface by benzoquinone. From the results in Figure 3c, we can assert that once the maximum performance of the eLac-C-SPE is reached, the laccase subsequently deposited does not significantly influence the performance of the device in terms of the amperometric response.

### Analytical Features

**Figure 4:**
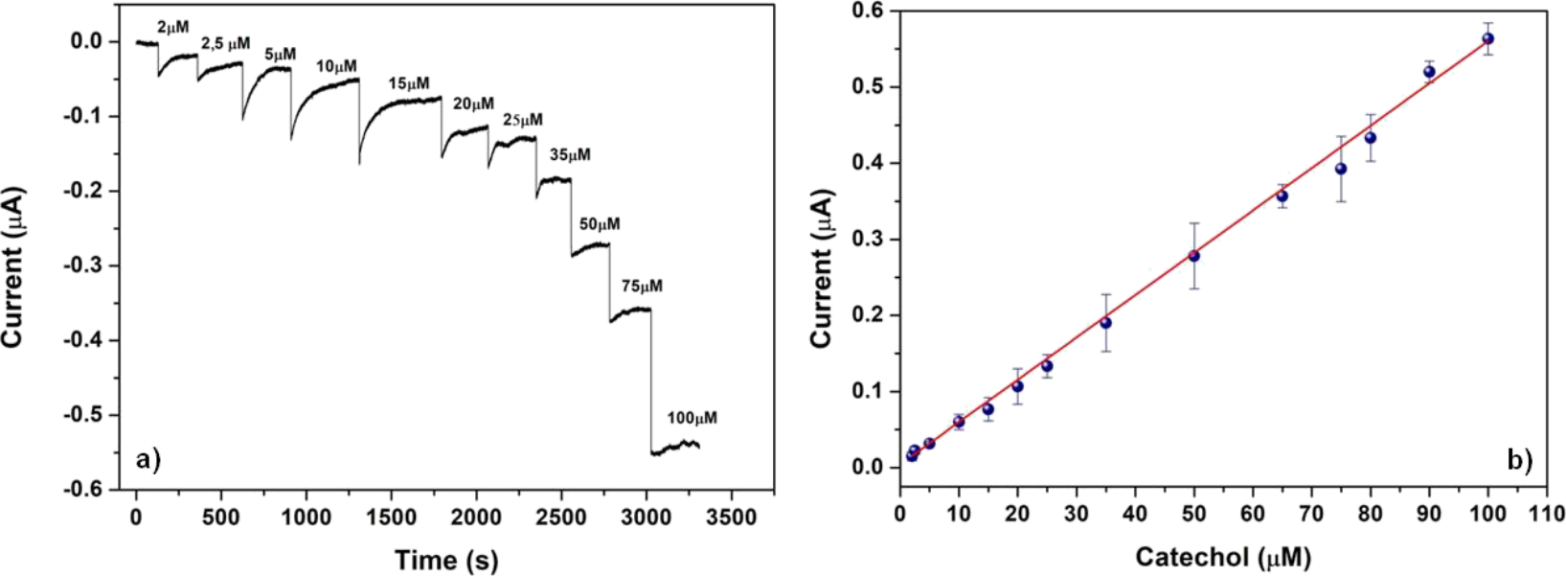
(a) eLac-C-SPE chronoamperogram at an applied potential of −0.03 V, showing the addition of increasing amounts of catechol and (b) corresponding calibration plot. Number of repetitions is *n* = 4. Measurement volume: 100 μL, 0.1 M acid citric/sodium citrate buffer at pH 4.5.

The detection capability of the eLac-C-SPEs has been tested toward catechol. The amperometric measurements have been performed at an applied potential of −0.03 V by dropping 100 μL of 0.1 M citric acid/sodium citrate buffer pH 4.5 on an SPE and incrementally adding increasing concentrations of catechol in the range from 2 to 100 μM, recording the current signals every 0.1 s. The chronoamperogram at increasing catechol concentrations is reported in Figure 4a. The current signal increases linearly with the catechol concentration, as described in Figure 4b, in which each measurement has been repeated four times on different electrodes produced in various batches. The average current value and the standard deviation of these measurements are reported vs catechol concentration in Figure 4b.

The calibration curve is given by *y* = 4.30 (±1.40) + 5.56 (±0.06)*x*, with an *R*^2^ = 0.998, where *y* is the measured current in nA, and *x* is the concentration in μM. The calibration curve returns a limit of detection, equal to 1.70 ± 0.05 μM, defined as 3.3*s*/*S*, where *s* is the standard deviation of the amperometric signals for three different measurements at the 5 μM concentration on the same electrode, and *S* is the slope of the calibration curve.^74^

### Working and Storage Stability Studies

**Figure 5:**
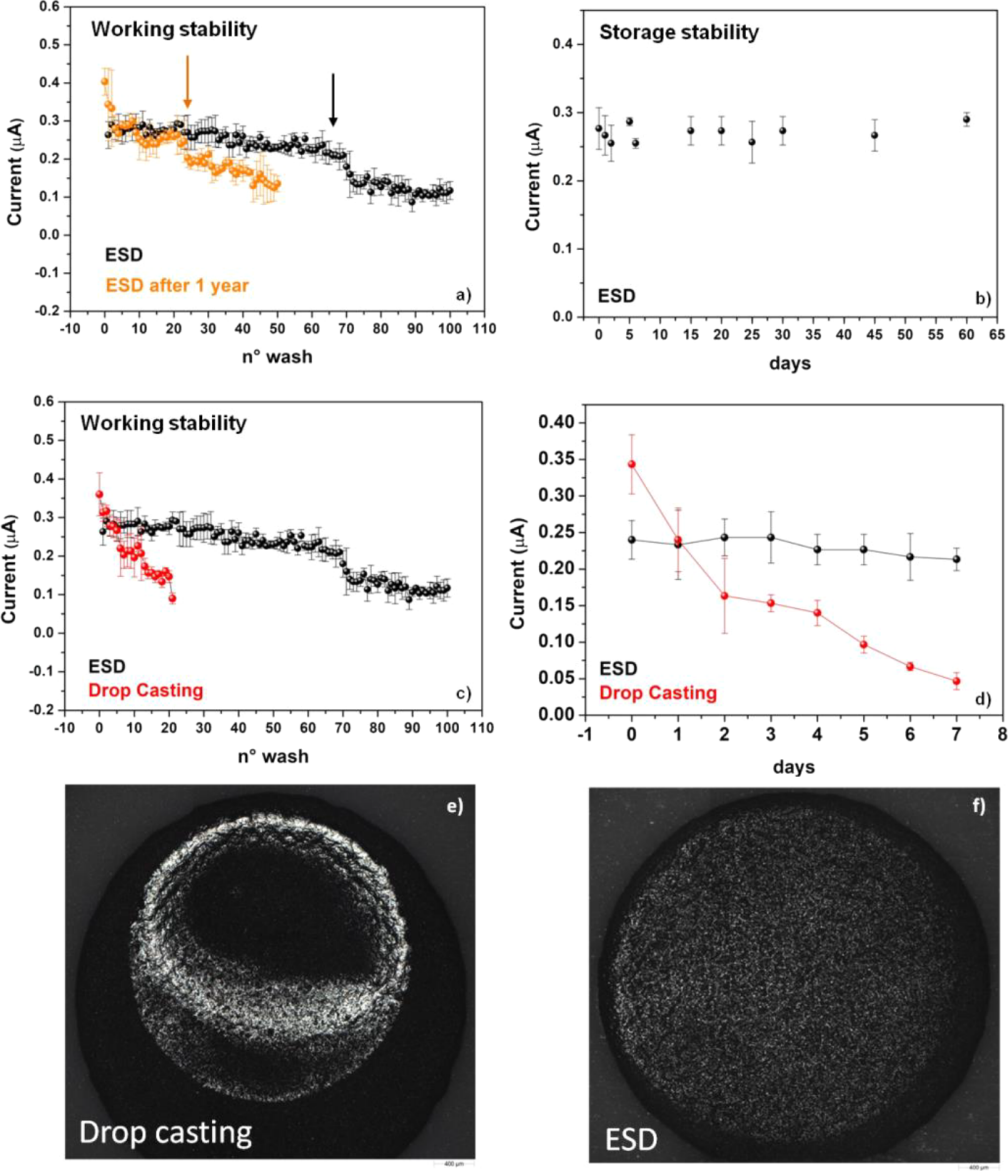
(a) Working stability of eLac-C-SPE fresh-made (black) and redeposited after one-year (orange) electrodes. (b) Storage stability of eLac-C-SPE. (c) Working stability of eLac-C-SPE fresh-made (black) and drop-casting (red) electrodes. (d) Working stability of the same eLac-C-SPE (black) and drop-casting (red) tested in subsequent days. For all the measurements, the applied potential is −0.03 V. Measurement volume is 100 μL of 0.1 M citric acid/sodium citrate buffer at pH 4.5 and 50 μM of catechol. Optical microscope image (Malvern Panalytical Morphologi 4-ID, magnification 2×) of (e) drop-casting deposit and (f) ESD deposit on the working electrode.

The operational stability of the eLac-C-SPEs has been investigated by repeating amperometric measurements on the same electrode in the presence of 50 μM catechol and alternating washes with 0.1 M citric acid/sodium citrate buffer at pH 4.5 between tests to check for enzyme leaching. The results shown in Figure 5a demonstrate a near 100% retainment of the activity up to 63 consecutive measurements within the statistical error, with a progressive decrease in the current signal to about 53% in 100 measurements. This gradual decrease in the current signal may be ascribed to the enzyme leaching out of the electrode. The present sensor exceeded the working stability achieved with the previous carbon black-modified sensors,^45^ which showed a resistance up to 25 washes. This result can be ascribed to a more uniform graphite surface on which the laccase is deposited compared to electrodes modified with carbon black nanoparticles by drop-casting, the latter resulting in the coffee-ring shape^51^ of the carbon black deposited on the graphite. Moreover, the laccase probably binds differently on the two substrates. Furthermore, after many washes, the removal of the carbon black, if not strongly bonded on the graphite, leads to a removal of the laccase layer deposited on it with a consequent reduction in performance. The absence of carbon black nanoparticles as well as any metallic nanostructure^75^ makes this new biosensor much more suitable for green disposal. Another outstanding result concerns the tests of working stability performed on reconditioned one-year-old electrodes to demonstrate the recycling nature of the new fabricated biosensor. For this test, a batch of three electrodes has been modified with electrosprayed laccase and put in storage for 1 year at ambient pressure and temperature remaining exposed to ambient light. After 1 year, the three electrodes have been subjected to another process of laccase deposition through ESI and then tested for working stability. The results shown by the orange dots in Figure 5a demonstrate maintenance of the activity toward the catechol detection at the same level as the fresh-made electrode up to a maximum of 20 measurements on the same electrode. After that, a gradual decrease up to 53% in activity is reached in 50 measurements. These results are extremely important in the perspective of the production of reusable and “environment-friendly” sensors, which ensure the reduction in pollution because of disposable sensors and guarantee the prolonged reuse over time of the same batch of sensors if they have not been used. Indeed, one may envisage that a producer of these types of biosensors could withdraw the product after 1 year from the production and subject it to another ESD process, putting it back on the market with comparable performance as it was just produced, with the aim of reducing pollution from disposable devices.

To evaluate the storage stability, eLac-C-SPEs deposited from different batches were preserved at room temperature and ambient pressure and light, and tested after a variable time from a few days to 2 months, with a solution of 50 μM catechol in a 0.1 M citric acid/sodium citrate buffer at pH 4.5 (Figure 5b). Each measurement was repeated three times. The results show excellent storage and photostability up to 2 months, achieved without any particular care in the storage of the electrodes.

To highlight the performance of the eLac-C-SPEs sensors, we compare the working stability with electrodes modified by drop-casting with the same quantities of the laccase enzyme (see Figure 5e, f). The measurements are performed on a batch of three electrodes modified using the drop-casting technique.

As shown in Figure 5c, even if the amperometric initial value of the SPEs modified using the drop-casting technique is higher compared to the one obtained by ESD, the stability along repeated washes is dramatically worse. The drop-casting SPEs lose the 78% of activity after almost 20 consecutive washes.

To compare these two different immobilization techniques, further measurements were carried out on consecutive days. In Figure 5d is shown the result obtained by carrying out one measurement per day on the same type of electrode modified through either ESD or drop-casting. The plotted value is the average of three measurements on three different electrodes for the two types of immobilization techniques performed once a day against 50 μM catechol in a volume of 100 μL of buffer at pH 4.5. It is clear that the drop-casting electrodes halve the activity already on the third day, while the ESD ones keep their activity unchanged until the seventh day. This may be ascribed to a weaker anchoring of the enzyme during adsorption by drop-casting than in the immobilization by ESD. The leaching of the enzyme is immediate and evident already on the second day on the drop-casting SPEs, while the ESD sensors are stable and can be reused the following days as if they were just manufactured. In other words, Figure 5d shows that the ESD sensor, after being used, can be left in the air for 24 h and gives the same signal of current, if reused the next day.

### Interferences Study and the Matrix Effect

**Figure 6:**
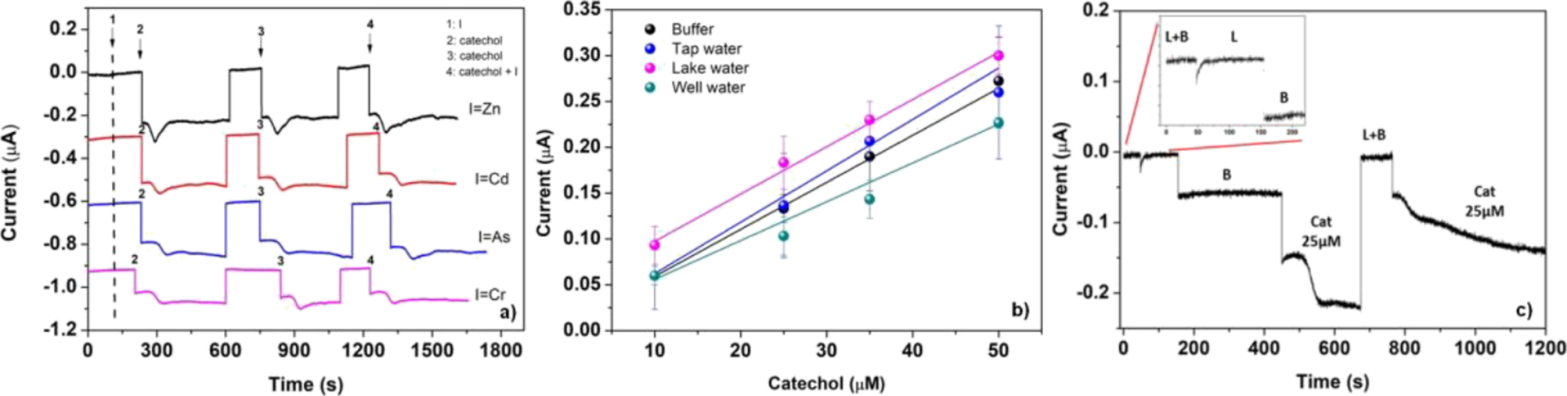
(a) Interferent study. The dashed line indicates the time at which the interferer was added. The times indicated as 2, 3, and 4 correspond to the addition of catechol 50 μM, catechol 50 μM, and catechol 50 μM + interference. For each of the consecutive measurements for the same interferent, the volume was 100 μL of 0.1 M sodium citrate buffer pH 4.5, (b) eLac-C-SPEs matrix effect. Applied potential −0.03 V, *n* = 3. Measurement volume: 100 μL. (c) Chronoamperogram of the eLac-C-SPE recorder for sequential additions of the lake water sample diluted (L + B) and not (L), buffer solution (B) and catechol 25 μM.

Laccase biosensors can suffer from interferents and/or electroactive species present in natural environmental samples of catechol contamination. Among the possible interferents present in real matrices as tap and surface waters as well as in well water, heavy metals were considered. In particular, the following limits of interfering species were tested: cadmium 5 μg/L, chrome 0.05 mg/L, arsenic 0.01 mg/L, and zinc 3 μg/L. These concentrations did not provide any significant response with respect to a corresponding catechol signal of 50 μM, both if added before and together with the catechol (Figure 6a). In the chronoamperogram, the measurement of 50 μM catechol performed between two consecutive measurements of interference, respectively, added before or together with the catechol shows that the sensor has no memory effect and is not affected in any measure by the presence of the metal ion (Figure 6a). With the aim to challenge the implemented biosensor in real samples, eLac-C-SPEs were tested by the analysis of 50 μM catechol in undrinkable tap water, lake, and well water (diluted 1:2 with 0.2 M sodium citrate buffer pH 4.5) using the standard addition method and the calibration curve from catechol standard solutions. As reported in Figure 6b, the obtained results confirm the absence of any matrix effect for tap water with slope ratios between the standard solution and real samples of 0.93. Even if lake water shows a slope equal to that of the standard solution, the higher current values can be probably attributed to other laccase substrates that are present in the sample and require further investigation. In the case of well water, the slope clearly differs from that of the standard solution, highlighting an interference behavior physically different from that of the lake water.

To test the possibility of using the same biosensor again in different matrices, the memory effect was analyzed using the sensor for the sequential analysis of the following samples: the lake water sample diluted 1:2 with 0.2 M sodium citrate buffer pH 4.5 and indicated as L + B in Figure 6c, then buffer solution (indicated as B), catechol, and finally L + B again. Figure 6c shows that the same value of current for L + B is reached after the removal of the buffer and catechol solution. This proves that the sensor can be reused several times by changing the matrix and always obtaining the same value of current, that is, it is not affected by the memory effect. As it is shown in the inset of Figure 6c, the lake water sample diluted or not with buffer gives the same current value, making the eLac-C-SPEs effectively usable in the real lake sample after calibration. Moreover, the sequential amperometric measurements of 25 μM catechol in 100 μL of B only or L + B on the same eLac-C-SPEs return the same values given in Figure 6b within the experimental uncertainty. This definitely proves that no memory effect exists.

## Conclusions

This study presents a low-cost, environment-friendly, efficient, and successful method for the construction of an electrochemical amperometric biosensor through the direct soft landing deposition of bioactive enzymes on the surface of C-SPE by ESD. The technique has been tested to fabricate a sensor based on the laccase enzyme from Trametes Versicolor. Laccase-based biosensors find applications in numerous fields like agri-food, pharmaceutical industry, environmental science for pollution monitoring, and environmental remediation. Thus, the development of a smart technique for the immobilization of this enzyme and for manufacturing reliable, reproducible, and portable sensors is paramount.

The ESD immobilization technique presented in this work is a one-step, environment-friendly method, allowing for the deposition of the biorecognition layer without using any additional chemicals (apart from a small amount of methyl alcohol easily replaceable with ethanol). The optimization of the deposition time, the solution to be sprayed, and the focusing geometry confirmed the importance of these parameters in determining the linearity, sensitivity, and signal stability of the biosensors. The results indicate an optimal deposition time of 15 min and the best-performing solution as the one with 20% methanol content. They also prove that the spot size of the deposit can be controlled using a focusing electrode to achieve the best fit of the deposited film to the working electrode area.

The most relevant result is the great performance in terms of reuse and storage. In particular, the possibility of reusing the just-made sensor 63 times consecutively and a one-year-old sensor subjected to redeposition for 20 consecutive times underlines the good anchoring of the enzyme, thanks to the ESD immobilization technique. This result is confirmed by the comparison with the drop-casting technique that fails to compete in terms of working stability.

The absence of additional chemicals during the immobilization phase and the peculiar performances in terms of reuse, time stability, and reconditioning of the sensor make both the process and the final product “*environmental-friendly and sustainable*.”

This ESD procedure can be extended to other types of enzymes or bioactive macromolecules with physicochemical characteristics suitable for a system based on electrochemical transduction. Therefore, it can find interesting and successful applications in biotechnology and bioengineering.

## Supporting Information Available

Electrospray deposition set up; experimental conditions for ambient soft-landing immobilization of laccase by electrospray; choice of the solvent and the study of the solvent effect; choice of the pH and the study of the optimal pH before and after ESD; effect of the electrospray ionization process; and choice of deposition time and focusing cone (PDF)
