# Supplementary material for: Fabrication of a New, Low-Cost, and Environment-Friendly Laccase-Based Biosensor by Electrospray Immobilization with Unprecedented Reuse and Storage Performances
Source: ACS Sustain Chem Eng. 2022 Jan 24;10(5):1888–98. doi: 10.1021/acssuschemeng.1c07604 (PMC8830555; doi:10.1021/acssuschemeng.1c07604)
Supplement: Supplementary file 1 — sc1c07604_si_001.pdf [file sc1c07604_si_001.pdf]

Supporting Information

Fabrication of a New, Low-Cost, and Environment-Friendly Laccase-Based Biosensor by Electrospray Immobilization with Unprecedented Reuse and Storage Performances

Mattea Carmen Castrovilli[*a], Emanuela Tempesta[b], Antonella Cartoni[c], Paolo Plescia[b], Paola Bolognesi[a], Jacopo Chiarinelli[a], Pietro Calandra[d], Nunzia Cicco[e], Maria Filomena Verrastro[f], Diego Centonze[g] , Ludovica Gullo[c], Alessandra del Giudice[c], Luciano Galantini[c] and Lorenzo Avaldi[a]

[a] *Istituto di Struttura della Materia-CNR, (ISM-CNR), Area della Ricerca di Roma 1, 00015, Monterotondo, Italy*

[b] *CNR-Institute of Environmental Geology and Geoengineering (CNR-IGAG), Area della Ricerca Roma1, Via Salaria km 29.300, 00015, Monterotondo, Italy*

[c] *Department of Chemistry, Sapienza University, P.le Aldo Moro 5, 00185 Roma, Italy*

[d] *CNR- Institute for the Study of Nanostructured Materials (CNR-ISMN), Area della Ricerca Roma1, Via Salaria km 29.300, 00015, Monterotondo), Italy*

[e] *CNR-Institute of Methodologies for Environmental Analysis, (CNR-IMAA), Contrada Santa Loja, Tito Scalo (PZ) 85050, Italy*

[f] *Istituto di Struttura della Materia-CNR, (ISM-CNR),Contrada Santa Loja, Tito Scalo (PZ) 85050, Italy*

[g] *Dipartimento di Scienze Agrarie, degli Alimenti e dell'Ambiente, Università degli Studi di Foggia, via Napoli, 25 - 71122, Foggia, Italy*

Corresponding author: Mattea Carmen Castrovilli matteacarmen.castrovilli@cnr.it

Number of pages: 10

Number of figures: 5

Number of tables: 4

1. Electrospray deposition set-up.

The procedure to identify the optimal operation conditions of the ESI source was as it follows: at lower voltages, the surface tension of the liquid overcomes the electric field, and the liquid flows drop by drop at the needle outlet. As the

voltage increases, the electric field becomes stronger and elongates the liquid meniscus into a cone whose apex extends into a jet (inset in Figure 1 main text). The onset voltage for the cone-jet mode of electrospray is given by the following equation [1,2,3]:

$$V \cong \sqrt{\frac{r_c \gamma \cos(49°)}{2\varepsilon_0} \ln\left(\frac{4D}{r_c}\right)} \tag{1}$$

where r_c is the radius of the needle, γ is surface tension of the solution, D is the distance between the needle and the counter electrode, and ε_0 is the permittivity of vacuum. The angle of 49° guarantees the equilibrium between an external electric field and a conical fluid surface, according to the model developed by Taylor [2]. In our set-up, the γ and D parameters, as well as the operating conditions with the focusing electrode, were optimized for the best performance in terms of spray stability and spot size of the deposit ($\Phi_{deposit}$). In detail, at r_c = 100 µm, the surface tension was optimized by testing different solutions described in Table S2 and the optimal distance D was found to be 14 mm. In order to deposit on a C-SPE working electrode area of about 4 mm diameter, the voltages of the needle and the focusing cone placed at a distance of 5 mm (Table S1) with respect to the SPE were set to 4.92 and 2 kV, respectively.

Table S1.

Geometrical sizes of the cones and diameter of the deposited film for each cone at d=5mm.
The geometric *h, Φ1* and *Φ2* parameters are described in Figure 1 main text.

Focusing Cone	h (mm)	Φ_1 (mm)	Φ_2 (mm)	$\Phi_{deposit}$ (mm)
C1	6	12	6	4.5
C2	6	13	6	4.5
C3	8	12	5	3.5
C4	6	9	5	3.5
C5	6	12	5	3.5

2. Experimental conditions for ambient soft landing immobilization of laccase by electrospray

It is known that the electrospray technique can hardly be performed using solutions containing the enzyme dissolved only in aqueous solvent, due to the high surface tension of water [4], or dissolved in the buffer, due to the amount of ions in the solution which require high voltages in order to obtain an excellent spray. For these reasons the effect of

two different solvents as methanol and ethanol in aqueous solution or in acid aqueous solution (0,01 % formic acid), that usually helps in the formation of a good nebulisation of the spray during the ESD process, has been tested (*Choice of the Solvent*). Furthermore the variation of the activity caused by the pH of solution (*Choice of pH*) and the electrospray ionization process itself (*Effect of Electrospray Ionization Process* in the main text) have been analysed. Finally the amount of laccase deposited on C-SPE can vary with the deposition time. By changing the focusing cone we can control the deposition area and find a good condition for the minimum deposited quantity of laccase which gives a detectable amperometric signal (*Choice of deposition time and focusing cone*).

2.1 Choice of the solvent: the study of the solvent effect

The effect of the solvent on the enzyme activity was studied by spectrophotometric measurements of four aqueous solutions containing different percentage of methanol, ethanol and formic acids. The absorbance measurements of syringaldazine (syr) at 530 nm showed that the best solution to be sprayed in order to preserve the enzyme activity was an acqueous one with the 20% of methanol. A control was run in parallel in the absence of organic solvent. The four solutions named *A*, *B*, *C* and *D* (Table S2) at laccase concentration of 2µg/µl have been prepared and stored for 30 minutes in order to mimic the condition of laccase in solution during the ESD process. After 30 minutes a volume of 30 µl of each solution, equal to the amount fluxed during the ESD process (flow rate of 1 µl/min for 30 min), has been taken. The activity of the enzyme has been measured by spectrophotometric analysis (*tA*, *tB*, *tC* and *tD* in Table S2) of the syringaldazine absorbance at 530 nm and 25°C. Syringaldazine is oxidised by laccase to the corresponding quinone producing water from oxygen molecules in solution; quinone production is monitored by light adsorption measurements at 530 nm. The buffer used is a 0.1 M pH 4.5 citric acid/sodium citrate solution (see section 2.2). The list of the prepared solutions is reported in Table S2.

Table S2.

List of working solutions tested to study the effect of different solvents on laccase activity.
The solutions A, B, C and D correspond to aqueous solutions with different percentages of organic solvent at laccase concentration of 2µg/µl. The tests tA, tB, tC and tD refer to the mixtures used in the cuvette for spectrophotometric analysis containing the respective solutions. The 'Blank' is the control one.

Solution	solvent percentage	Test	Test Solutions
A	20% MetOH	tA	30 µl solution A / 1320 µl Buffer / 150 µl syr
B	20% EtOH	tB	30 µl solution B / 1320 µl Buffer / 150 µl syr
C	20% MetOH/0,01% AcForm	tC	30 µl solution C / 1320 µl Buffer / 150 µl syr
D	20% EtOH/0,01% AcForm	tD	30 µl solution D / 1320 µl Buffer / 150 µl syr
Blank	60% Buffer/40% H_2O	tBlank	30 µl Blank / 1320 µl Buffer / 150 µl syr

Three spectrophotometric measurements have been performed for each solutions listed in Table S2 and the average results are reported in Figure S1.

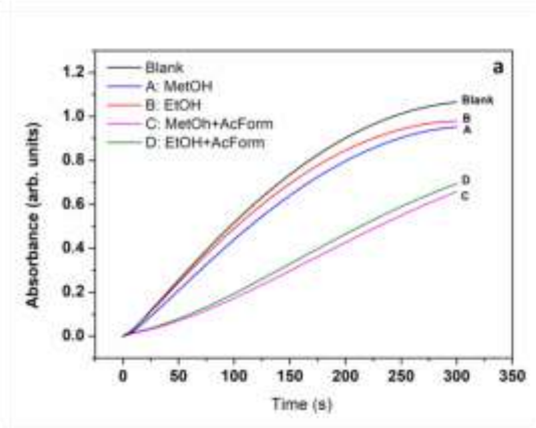

Figure S1. Comparison of the absorbance curves of solution A (blue curve), B (red curve), C (yellow curve), D (green curve) and Blank (black curve) measured at 530 nm according to the procedure indicated in the text.

The curves *C* and *D* suggest that even if the formic acid is usually used in ESI to increase the electroconductivity of the solution, its use as a protonating agent in the case of the laccase enzyme is not recommended because it reduces the activity of the enzyme in solution. A 20% solution of methanol or ethanol, instead, has a negligible influence on the activity as shown by the curves A and B.

These results are in agreement with the ones by Leonowicz and Gzywnowicz [5] for laccase from Trametes Versicolor, who demonstrated no variation in syringaldazine absorption up to 25% of ethanol concentration in solution. They observed a first decrease in laccase activity in solutions containing an amount between 27.5% and 80% of ethanol or 50% of methanol. In particular they found a loss of activity of 50% with a 50% concentration of methanol in the reaction mixture. This is consistent with our results of a 17% loss of activity for a solution with a 20% methanol

concentration obtained by comparing the slopes of curves *A* and *Blank* in the steady state region [6]. On the basis of these results and having observed a greater stability of the electrospray process with solution *A*, methanol has been chosen as a co-solvent of water for all the other tests.

2.2 Choice of the pH: the study of optimal pH before and after ESD

The crucial role of pH on enzymatic activity is well-known. The optimal pH value is characteristic of each enzyme and it depends on the chemical environment, temperature and enzyme stability in acid and alkaline neighbourhood. According to literature [7] the most commonly used buffer for laccase Trametes Versicolor of 0.5 U/mg is a citric acid/sodium citrate 0.1 M solution. The optimal pH value was searched within the range 3.5 - 6 using spectrophotometric measurements of the syringaldazine absorbance at 530 nm before deposition and by amperometric measurements of the current produced by the red-ox reaction of the catechol catalyzed by the electrosprayed laccase on C-SPE after the deposition. The laccase sensing mechanism for catechol detection is based on the electrocatalysis of catechol oxidation to its corresponding 1,2 benzoquinone, which is coupled with the electrocatalytic reduction of dioxygen to water on the working electrode surface. *Before* ESD, in a systematic exploration, six different solutions of citric acid/sodium citrate 0.1 M were prepared at pH values between 3.5 and 6 (see Figure S2a). The absorbance curves were measured by repeating test tBlank in Table S2 with the six buffer solutions to establish the optimal pH for laccase. Three spectrophotometric measurements have been performed for each pH value and their average is shown in Figure S2a. Then, three depositions of 30 minutes each were performed on three C-SPEs with the set-up shown in Figure 1. of the manuscript. The so modified electrodes were tested by amperometric measurements, to analyse the activity of the enzyme *after* the ESD, using the same buffer solutions (citric acid/sodium citrate at 0.1 M) at room temperature.

<u>Before ESD</u>

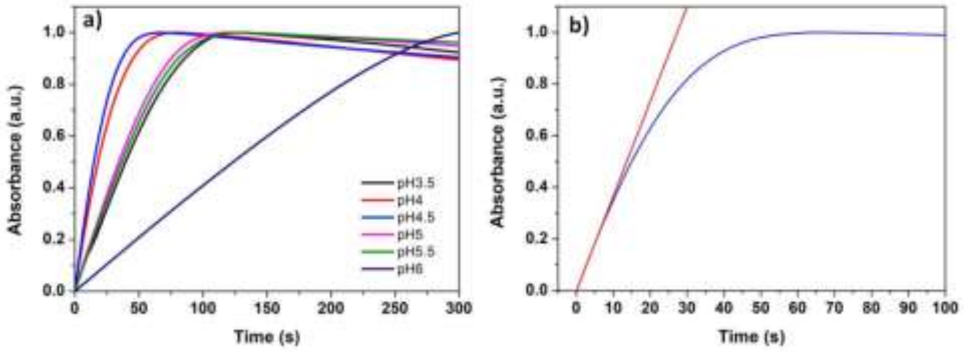

Figure S2. Absorbance values for the syringaldazine oxidation catalyzed by laccase, at different pH values (a). Determination of the initial rate of syringaldazine oxidation reaction at pH=4.5 (b).

In Figure S2b the slope of the absorbance *A* versus time at the beginning of the reaction represents the initial rate v_0 of the reaction for pH of 4.5.

$$v_0 = \frac{\Delta A}{\Delta t} \qquad (2)$$

For t_0=0, the initial rate is v_0=0.047 ΔA/t for the curve at pH=4.5. To correlate the v_0 to the variation of concentration in time the Lambert-Beer law $A = \varepsilon c \ell$ is used, where ε is the molar extinction coefficient of syringaldazine, *c* the concentration and ℓ the optical path equal to 1 cm.

$$v_0 = \frac{\Delta A}{\Delta t} = \frac{\epsilon \Delta c \ell}{\Delta t} \qquad (3)$$

By rearranging eq. 3 :

$$\frac{\Delta A}{\Delta t\, \epsilon \ell} = \frac{\Delta c}{\Delta t} \qquad (4)$$

thus if ε=65 $mM^{-1}cm^{-1}$ [8] then Δc/Δt=0.72 (μmol/L)/s at pH=4.5. The calculated $v_0 = \Delta A/\Delta t$ for each pH value are shown in Figure S3a, while the corresponding variations of concentration per second ($\Delta c/\Delta t$) are collected in Table S3. The results clearly show that the optimal pH value for the enzymatic reaction in solution is 4.5.

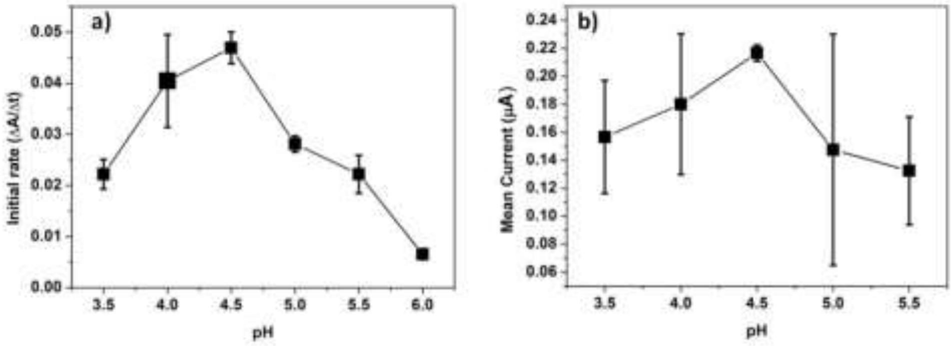

Figure S3. Initial rate for different pH values. The error is the standard deviation calculated on the average of 3 measurements for each pH value (a). Amperometric measurements of the laccase-catechol system, for laccase deposited on C-SPE. The shown values are the average of four amperometric measurements made for each pH value at cathecol concentration of 30 μM (b).

After ESD

Since it is possible that, during the electrospray process, the enzyme can suffers: (i) inactivation inside the ES capillary due to the electrochemical reactions, (ii) inactivation as a result of reaction with corona products in the gas phase, (iii) inactivation as a result of impact with the target electrode [9], and therefore be deposited in conformations different from those in solution, the study of the activity versus the pH was also carried out by amperometric measurements on laccase deposited for 30 minutes on C-SPE at catechol concentration of 30µM (Figure S3b). The data in Figure S3b do not show significant differences and therefore the use of the optimal pH of the native enzyme has been preferred.

Table S3.

Variation of the concentration calculated as µM/s.
The values are derived from the absorbance curves, which are the average of three measurements for each pH value.

pH	Δc/Δt
3.5	0.34 ± 0.04
4	0.62 ± 0.14
4.5	0.72 ± 0.05
5	0.43 ± 0.02
5.5	0.34 ± 0.05
6	0.10 ± 0.01

2.3 Effect of Electrospray Ionization process

The results of the siringaldazyne assay performed on test solution *tA* and on laccase dissolved after deposition (without cone) of solution *A* are shown in Figure S4 where the absorbance curves measured in the two cases are compared. The change of slope of the two curves in the steady state region shows that laccase activity after ESD immobilization is equal to 70% of that from starting enzymatic solution.

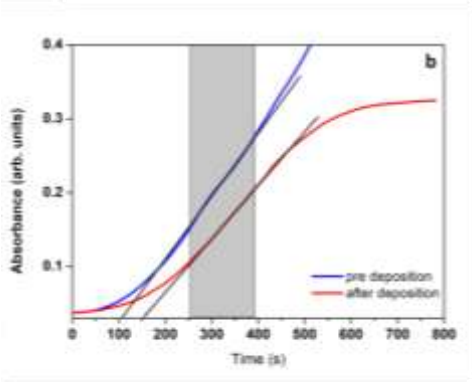

Figure S4. Comparison of the absorbance measured at 530 nm for the solution A (blue curve) and for the laccase dissolved after deposition by ESD (red curve). The linear fits (black lines) in the steady state (grey box) are also shown.

2.4 Choice of deposition time and focusing cone

To quantify the fraction of material intercepted by the cone, the amount of deposited enzyme has been evaluated by substituting the target with the resonator of a quartz crystal microbalance (QCM).

Our custom QCM is composed by two gold electrodes with a diameter of 6 mm obtained by vapour deposition on a quartz crystal disk of 14 mm diameter [10,11] and a resonance frequency f0 = 10 MHz. The variation of the resonance frequency is proportional to the amount of deposited material [12]. However, it also depends on the viscoelastic properties of the deposited material, the adhesion on the gold material and among different layers. As such, the response frequency versus mass had to be calibrated specifically for laccase.

To calibrate the QCM response, controlled amounts of laccase were deposited by dropcasting on the resonator. Then the QCM has been used to measure the amount of the deposited laccase for each focusing electrode (Table S1). Three independent depositions for each cone were done. Then, in order to test the linearity in the ESD rate the amount of the deposited laccase was measured versus the deposition time, with three measurements at each time.

Considering that the concentration of the sprayed solution is 2 µg/µl and the spraying rate is 1 µl/min, the expected 'nominal' amount of deposited material can be easily calculated and compared to the measured one through the procedure reported in the section devoted to the study of the deposition time in the manuscript. The fraction of deposited material, depending on the focusing electrode varies, from 45 to 21 % of the sprayed material.

To evaluate this result one can compare these quantities with the ones calculated for an hypothetical direct deposition on a mask with a hole of diameter $\phi_{deposit}$ equal to the one of the spot obtained with a focusing electrode (see Table S1). The amount of deposited material can be calculated by the following equation (5):

$$mass = Vol \cdot c \cdot (\phi_{\text{deposit}}/\phi_0)^2 \qquad (5)$$

where *Vol* is the total volume of solution sprayed, *c* is enzyme concentration, and ϕ_0 = 15 mm is the diameter of the deposited film at 14 mm distance from the spray needle without any focusing electrodes. In Table S4 the amount of enzyme deposited in 20 min by the use of conical electrodes (Figure S5) or in the hypothesis of using a mask to achieve the same spot size (eq. 5) are reported. The comparison clearly shows a gain in the amount of deposited material of a factor between 4 and 6 thanks to the focusing electrode.

Table S4.

Amount (μg) of deposited enzyme after 20 min at flow rate of 1 μl/min and a concentration of 2 μg/μl by using focusing conical electrodes or in the hypothesis to use a mask (eq.5). The percentage of the deposited enzyme with respect to the nominal amount (40 μg) is reported in bracket.

**configuration**	**Cone C1**	**Cone C2**	**Cone C3**	**Cone C4**	**Cone C5**
Focusing electrode	18.2 (45%)	13.2 (33%)	11.8 (30%)	12.5 (31%)	8.4 (21%)
Hypothetical mask	3.6 (9%)	3.6 (9%)	2.2(6%)	2.2 (6%)	2.2(6%)

In the calculation, possible focusing effects introduced by the material of the mask and the ions deposited on it have not been considered [13].

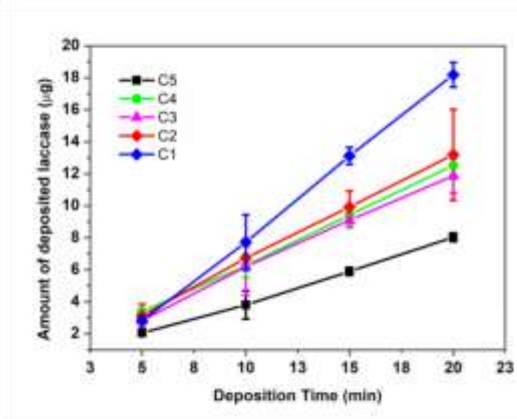

Figure S5. Amount of deposited laccase on the QCM electrode using the conical electrodes labelled Ci (i=1,5) in Table S1 to focus the spray. The spray solution A in Table S2 has been used. The voltage settings and geometrical parameters for the deposition are the ones in Figure 1 in the main text.

[1] Kavadiya, S.; Biswas, P. Electrospray deposition of biomolecules: Applications, challenges, and recommendations. *Journal of Aerosol Science* **2018**, *125*, 182–207. DOI: 10.1016/j.jaerosci.2018.04.009.

[2] Taylor, G. Disintegration of water drops in an electric field. Proceedings of the Royal Society of London A: Mathematical. *Physical and Engineering Sciences* **1964**, *280*, 383–397. DOI: 10.1098/rspa.1964.0151.

[3] Cloupeau, M.; Prunet-Foch, B. Electrostatic spraying of liquids: Main functioning modes. *Journal of Electrostatics* **1990**, *25*, 165–184. DOI: 10.1016/0304-3886(90)90025-Q.

[4] Borra, J.; Tombette, Y.; Ehouarn, P. Influence of electric field profile and polarity on the mode of EHDA related to electric discharge regimes. *Journal of Aerosol Science* **1999**, *30*, 913–925. DOI: 10.1016/S0021-8502(98)00779-4.

[5] Leonowicz, A.; Grzywnowicz, K. Quantitative estimation of laccase forms in some white-rot fungi using syringaldazine as a substrate. *Enzyme. Microb. Technol*. **1981**, *3*, 55-57. DOI: 10.1016/0141-0229(81)90036-3

[6] Briggs, G. E.; Haldane, J. B. A Note on the Kinetics of Enzyme Action. *Biochemical Journal* **1925**, *19*, 338-339. DOI: 10.1042/bj0190338.

[7] Das, P.; Barbora, L.; Das, M.; Goswami, P. Highly sensitive and stable laccase based amperometric biosensor developed on nano-composite matrix for detecting pyrocatechol in environmental samples. *Sensors and Actuators B* **2014**, *192*, 737-744. DOI: 10.1016/j.snb.2013.11.021.

[8] Manole, A.; Herea, D.; Chiriac, H.; Melnig, V. Laccase activity determination. *Scientific Annals of Alexandru Ioan Cuza din Iaşi University,* Biomaterials in Biophysics Medical Physics and Ecology **2008**, 17–24. http://www2.phys.uaic.ro/labs/comb/analele%20stintifice/2008/3_manole%208pag.pdf (accessed 2018-02-15).

[9] Morozov, V. N.; Morozova, T. Y. Electrospray Deposition as a Method To Fabricate Functionally Active Protein Films. *Anal. Chem*. **1999**, *71*(7), 1415–1420. DOI: 10.1021/ac9808775.

[10] Macagnano, A.; Zampetti, E.; Pistillo, B. R.; Pantalei, S.; Sgreccia, E.; Paolesse, R.; d'Agostino, R. Double layer sensors mimic olfactive perception: A case study. *Thin Solid Films* **2008**, *516*, 7857-7865. DOI: 10.1016/j.tsf.2008.05.025.

[11] Bearzotti, A.; Macagnano, A.; Papa, P.; Venditti, I. A study of a QCM sensor based on pentacene for the detection of BTX vapors in air. *Sensors and Actuators B: Chemical* **2017**, *240*,1160-1164. DOI: 10.1016/j.snb.2016.09.097.

[12] Wasilewski, T.; Szulczynski, B.; Kamysz, W.; Gebicki, J.; Namiesnik, J. Evaluation of Three Peptide Immobilization Techniques on a QCM Surface Related to Acetaldehyde Responses in the Gas Phase. *Sensors* **2018**, *18*, No. 3942. DOI: 10.3390/s18113942.

[13] Morozov, V. N.; Morozova, T. Y. Electrospray Deposition as a Method for Mass Fabrication of Mono- and Multicomponent Microarrays of Biological and Biologically Active Substances. *Anal. Chem*. **1999**, *71*(15), 3110–3117. DOI: 10.1021/ac981412h.
